# Do public officials exhibit social class biases when they handle casework? Evidence from multiple correspondence experiments

**DOI:** 10.1371/journal.pone.0214244

**Published:** 2019-03-27

**Authors:** Nicholas Carnes, John Holbein

**Affiliations:** 1 Sanford School of Public Policy, Duke University, Durham, NC, United States of America; 2 Department of Political Science, Brigham Young University, Provo, UT, United States of America; Sogang University (South Korea), REPUBLIC OF KOREA

## Abstract

Are public officials more responsive to requests from affluent or poor constituents? A growing body of evidence suggests that lawmakers are more responsive to the rich when they craft policy. However, some scholars theorize that officials also exhibit a corresponding bias *in favor of the poor* when they handle casework, essentially giving policy to the rich and services to the poor. In this paper, we test this casework prediction using four experiments in which confederates sent simple requests to state or local officials. In each, our confederates’ reported social classes were randomly assigned and signaled with a brief introductory statement mentioning the sender’s occupation or economic situation. Across our samples, we find precisely-estimated null effects of social class biases: the officials we studied were equally likely to respond regardless of the constituent’s class. These findings raise doubts about whether casework is really a class-biased process.

## Introduction

A growing body of evidence suggests that politicians in the United States are far more responsive to the preferences of affluent Americans than to the views of middle- and working-class citizens. When legislators cast roll call votes, their choices are more strongly associated with the views of higher-income citizens than with those of the less fortunate [1, 2]. When laws change, they tend to move toward outcomes that more privileged Americans favor, regardless of what less affluent citizens want [3, 4, 5, 6]. (But see [7] and [8].)

If the privileged tend to get their way in the legislative process, do they also tend to get their way in the many other important stages of the political process? Are the federal agencies that implement new laws more responsive to the views of the rich, too? Do the street-level bureaucrats who carry out the day-to-day work of federal, state, and local governments exhibit the same kind of unequal responsiveness scholars have observed among members of Congress? When citizens reach out to public officials for help with day-to-day needs—with *casework* requests—are the rich favored in that process, too? In short, just how deep does the unequal responsiveness scholars have observed in the passage of state and federal laws really run?

Direct constituent services may be one site where the less fortunate are actually *better* represented than the affluent. One emerging school of thought argues that more affluent Americans tend to have more clear and forceful policy preferences, while less affluent Americans tend to have more concerns about the basic demands of day-to-day life. In this view, politicians seeking to keep their constituents happy might essentially give policy to the affluent and services to the less fortunate, giving rise to a system in which the privileged are disproportionately influential with respect to legislation, while the less privileged are disproportionately served through constituent service.

Do the less fortunate really receive preferential treatment when they put in constituent service or casework requests? Or does the unequal responsiveness scholars have observed in federal and state lawmaking extend to casework, too? To find out, we conducted four randomized-control trials on state and local public officials. In each experiment, we sent the kinds of requests that officials frequently receive from constituents seeking their help. Experiments 1 and 2 focused on *state legislators*, a group of politicians that collectively has a powerful influence over distributional outcomes and that has been found to be unequally responsive when it comes to legislation [5, 9]. In these experiments, a confederate emailed legislators with simple requests—the first asking for help registering to vote and the second requesting a brief in-person meeting—and randomly varied the confederate’s stated occupation. Experiment 3 focused on *public school principals*, consequential local “street-level bureaucrats” who also have frequent contact with citizens [10]. In this larger-scale experiment, confederates asked principals for information on school music and art programs. Finally, Experiment 4 focused on *mayors* in the United States—important elected officials who influence public policy and constituent-government interactions in important ways.

In all of these experiments, we randomly varied whether public officials received correspondence from a more or less affluent person by varying the individual’s stated occupation (Experiments 1, 2, and 4) or biographical narrative (Experiment 3). This follows the approach of similar recent audit studies on racial, ethnic, age, and gender bias [10, 11, 12, 13, 14, 15], which typically use relatively small manipulations (e.g. changing the name of the sender).

Whereas other recent audit studies find clear evidence that politicians discriminate on the basis of race, ethnicity, age, and religion when responding to constituent requests, we find no evidence of an analogous social class bias in any of our experiments. In Experiments 1 and 2, state lawmakers were just as likely to grant our confederate’s requests regardless of whether they perceived the confederate as an HR professional or a dishwasher in a restaurant. In Experiment 3, public school principals were just as likely to respond to request for information from a citizen who described financial hardships and a citizen who did not. Experiment 4—which magnified the class divisions even further by making comparisons between jobs at the top of the income distribution to those at the bottom—likewise found (precisely-estimated) non-significant and substantively trivial differences. When pooled together, the 95% confidence interval from these experiments allow us to confidently rule out differences in favor of the more affluent confederate as small as 1.32 percentage points—an effect that is statistically and substantively smaller than previous audit studies of race, ethnicity, age, and religion.

These null effects represent a significant departure, both from the growing research on demographic biases in political casework, and from the research on unequal responsiveness among politicians in the United States. The non-differences we observe do not appear to be the result of a lack of statistical power, nor can we attribute them to the level or type of office studied or the type of social class manipulation being used. Routine casework simply may not be as biased towards the rich as other aspects of the government, as biased towards the poor as scholars theorize, or as biased along social class lines as it is on other demographic dimensions.

## Past research

Most recent work on constituent services has found clear evidence that politicians prioritize requests from some social groups over others. The basic theoretical logic is straightforward: politicians usually receive more requests than they can answer, so they prioritize requests from social groups that could benefit them or that they have some personal connection to. In one of the most notable recent studies on this topic, Butler and Broockman emailed roughly 5,000 state legislators across the country posing as a constituent who wanted help registering to vote [12]. They signed half the emails “Jake Mueller,” a name they had previously determined was almost always the name of a white person, and signed the other half “DeShawn Jackson,” a name that was almost always that of a black person (based on data from the U.S. Census). DeShawn received significantly fewer responses (the gap observed between the two treatment conditions was 5.1 percentage points, *p* = 0.04). Even when legislators had strategic incentives to help the constituent—when the citizen added that he was a member of the legislator’s party—lawmakers often discriminated on the basis of race, suggesting the bias was a matter of more than just electoral strategy. Other audit studies have reached similar conclusions: officeholders have been found to be less responsive to requests from Hispanics, Muslims, and the elderly.

Do politicians similarly prioritize the rich—or the poor—when answering casework requests? There are reasons to expect that they might, as the last section noted. On the other hand, it is also possible that other traits matter more, or that officeholders do not prioritize requests based on the sender’s social class. The very act of contacting a public official might convey to him or her that a constituent is more politically engaged, thereby negating any assumptions the politician might have made about engagement based on the constituent’s social class.

To date, experimental research on this point has been rare. We know of just one study on this topic: Butler finds that mayors in the United States are more likely to answer an email from an out-of-town citizen who claimed to be a homeowner and who asked about the town’s advanced placement programs than an out-of-town citizen who claimed to be a renter and who asked whether the town’s high schools offered a free lunch program [16].

The Butler study illustrates a difficulty in looking for class discrimination in constituent services: establishing the actual treatment itself. Whereas studies of racial/ethnic discrimination can relatively cleanly alter the racial identity of the sender, with a small change to the sender’s name, the same cannot be said with class as names break less cleanly on class lines. In this paper we use two kinds of class manipulations—one that builds on Butler’s approach and directly tells the subject that the sender is on a free-reduced price lunch program, and another that mentions the sender’s occupation. Our goal here is to be as comprehensive as possible—to use multiple ways of signaling class to see if they yield similar results.

## Materials and methods

To determine whether state and local officials exhibit class bias in their responses to casework requests, we conducted four audit experiments, which we describe below. In all of our experiments, we randomized whether confederates portrayed themselves as more or less affluent, a point we conveyed in different ways across the different studies (since no single approach to conveying social class in written correspondence is perfect). Confederates gave informed consent prior to the start of the study and were fully informed about the research question, study methodology, and findings. Once we ran the experiments, we compared the response rates in each pair of treatment conditions with *t*-tests and regression models. This study was approved by the Duke University IRB ([A0779] Class and Legislative Correspondence). Informed consent for the legislators, principals, and mayors was waived by Duke University’s Internal Review Board. We obtained informed consent from the state residents who served as confederates in the study.

### Experiments 1 and 2: State legislators

In Experiments 1 and 2, our sample consisted of all state legislators in North Carolina in 2012 and 2013. In both, we partnered with a confederate who was an actual North Carolina voter and whose job duties entailed both washing dishes in a restaurant and working as an HR professional. In each experiment, the confederate emailed every member of the North Carolina state legislature with a simple request, and began each email by mentioning his name and a randomly-assigned occupation: “My name is Joey, and I’m [a dishwasher / an HR professional].”

We expected these two occupations to convey very different information to the recipient. In general, occupations provide a strong signal of a person’s position in a society’s economic or social class structure—as Donald Matthews put it, what we do for a living is “[p]robably the most important single criterion for social ranking in the United States” [17]. These two occupations, moreover, should evoke very different expectations about the sender’s income, social status, and so on [18]. According to the BLS, a dishwasher’s average salary in 2010 was $18,930, putting the occupation around the 20th percentile of the individual income distribution; whereas an HR manager’s average salary was $52,690 per year, placing them around the 60th percentile. (We use even more sharply distinct occupations in Experiment 4 below.) When legislators saw “dishwasher” vs. “HR manager,” there are strong reasons for them to perceive clear differences in the affluence of the sender.

Following Butler and Broockman [12], in Experiment 1, we worked with our confederate to email every member of the North Carolina General Assembly to ask for *information about registering to vote*. (Requests like these are extremely routine, and North Carolina legislators do not share or “pool” staff, so we did not have any reason to expect that staffers or legislators would discuss the requests with one another, nor did we ever encounter any evidence that email recipients were aware that their behavior was being studied.) The body of the email was modeled after previous audit studies (see Table A in the S1 Appendix). 84 legislators received an email from the dishwasher, and 86 received an email from the HR professional. Our randomization yielded nearly perfect balance on other observable characteristics, like the legislator’s race and gender and legislative background.

Six months later (in early 2013), we ran Experiment 2, again working with our confederate. We expected that this would be long enough that there was little chance that legislators or staffers would remember the first email and, indeed, none who responded to the email in the second experiment mentioned any previous correspondence. We also used a different email address, in case some legislators maintained databases or email archives. Here we emailed state legislators to ask for something slightly costlier: *time to meet*. With our help, our confederate emailed every state legislator to ask to meet or speak on the phone about a recent voter ID bill. The subject line read, “meeting to discuss voter registration” (for the text of this email see Table B in the S1 Appendix). This request was substantially costlier than the request for simple information [19], which we hoped would allow us to better detect any social class biases that might exist.

With both experiments, we recorded politicians’ responses for two weeks, including the length of the response, whether it was helpful (meaning it clearly explained how to register to vote, or offered to set up a meeting), and whether the email was signed by a staffer or the legislator. If state lawmakers prioritized requests from more affluent citizens, Experiments 1 and 2 should have detected it.

To check that imbalances in confounding variables weren’t driving these results, we also estimated logistic regression models that controlled for several additional factors. When we randomized our sample, the resulting treatment groups were well-balanced on a wide range of observable variables. Still, as a simple robustness check, we estimated models which controlled for the legislator’s previous occupation, party, and chamber; whether the legislator was on the state elections committee; the legislator’s race and gender; how long the legislator had been in office; whether the legislator was a party or committee leader; and the number of staffers the legislator employed. None of these controls changed our findings presented below.

### Experiment 3: Public school principals

Because these experiments had relatively small samples, in Experiment 3, we focused on a larger sample of public officials: public school principals in North Carolina and Kentucky. School principals are what Lipsky refers to as street-level bureaucrats, local government officials who receive numerous casework requests and make many consequential decisions. Unlike state legislators, principals are not subject to direct elections, which significantly diminishes any electoral incentives that might compel them to respond more to the rich or the poor [20]. In that sense, principals are useful because they provide a window into the non-electoral (e.g., social or personal) incentives public officials might have to prioritize the rich or the poor.

We first randomly selected 719 principals, then randomly assigned half to receive a *less affluent* treatment email and half to receive a *more affluent* email. The subject of both emails was “Music and Art Programs,” and like the first two experiments, we recruited confederates who were state residents and who were interested in learning more about the subject at hand.

In addition to increasing our sample size and statistical power, another goal of Experiment 3 was to make the social class signals even more pronounced. Rather than just signaling class by mentioning a job, in the *less affluent* treatment, the email included a paragraph that described how the confederate had struggled financially, had been on food stamps, and had a son who was on free/reduced price lunches at school (see Table C in the S1 Appendix)—clear markers of a less affluent person. In the *more affluent* treatment, we simply omitted this paragraph (as it is hard to imagine analogous language that would convey affluence and that would not sound artificial—e.g., “my child is *not* on free/reduce price lunches.”) As in Experiments 1 and 2, we recorded how the officials in question responded for two weeks.

### Experiment 4: Mayors

In a final experiment, we conducted a similar audit of a random sample of just over 3,400 mayors in the United States. Following other recent studies of how mayors respond to constituent service requests (e.g., Butler 2014), our sample was drawn from the American Municipal Officials Survey (AMOS)—which consists of the largest database of elected municipal officials [21]. We focus on mayors given their direct role in many constituent services.

As in Experiment 3, our goal in this experiment was to increase the statistical power of our test and, more importantly, to increase the treatment intensity, both by increasing the apparent economic differences between our more and less affluent treatment categories, and by increasing the fraction of the sample exposed to treatment (a commonly used experimental technique to detect small effect sizes; e.g., [22]). We assigned just under 60% of the sample to the more affluent treatment condition given theoretical predictions of bias towards this group. One potential criticism of Experiments 1 and 2 is that the differences between a dishwasher and an H.R. professional (i.e. a comparison of someone at the 20th and the 60th percentiles) might not be large enough for elected officials to notice, or that elected officials might think both groups were in the middle- to lower-classes, not the *upper* class. To increase the salience of class via our occupational treatments, we selected three less affluent professions and four even more affluent professions: mayors were randomly assigned to receive an email from a grocery store clerk ($22,130 per year), an auto mechanic ($29,700), or a farm worker ($25,070)—occupations in the bottom decile of the income distribution—or from a pharmacist ($121,710), a dentist ($180,010), a software developer ($111,780), or a personal finance advisor ($124,140)—occupations in the top 5% of the income distribution. These occupations are far more socially and economically distinct than the occupations used in Experiments 1 and 2.

In Experiment 4, we sent emails posing as hypothetical constituents requesting information about who to call before digging for a landscaping project. So as to not signal homeownership, all emails simply stated that the constituent was “planning to do some landscaping work,” a vague phrase that might denote anything from home improvement to needed repair work or even a second job. This common request is within the purview of local officials, like mayors, and is a constituent request that takes little time to answer. It also is a request that one from various class backgrounds might feasibly ask. (For the text, see Table D in the S1 Appendix.)

## Results

**Fig 1:**
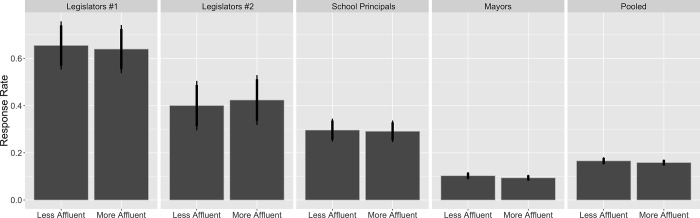
Response rates by treatment condition. Each panel reports the results of one experiment. Bars plot average response rates with corresponding 90% (wide) and 95% (narrow) confidence intervals. N = 170 (first), N = 170 (second), N = 719 (third), N = 3433 (fourth), N = 4492 (fifth; pooled).

Across our four experiments, our confederates received responses from 16.2% of public officials (64.7% in Experiment 1, 41.2% in 2, 29.4% in 3, and 9.8% in 4). Experiment 1’s overall response rate was only slightly higher than (and not statistically distinct from) the response rate in Butler and Broockman’s national study (57%) [12]. The decline in response rates in lower levels of government is consistent with our expectations, especially given that local officials are less likely to have staffs that help respond to emails. Fig 1 plots the response rate broken down by our class treatments, with the pooled estimates shown on the far right.

In all four experiments, the officials we studied were equally likely to respond to our confederates’ emails regardless of how they described their social classes. In Experiment 1, 64.0% of North Carolina legislators (or their staffs) responded to a request for voter registration information from a white-collar professional, and 65.5% responded to a request from a working-class citizen (Fig 1, first panel). This difference was substantively small and not statistically significant (*p* = 0.84). In Experiment 2, the response rates were 40.0% for a white-collar professional requesting a meeting and 42.4% for a blue-collar worker (Fig 1, second panel). Again, this difference was small, and not close to being statistically significant (*p* = 0.76). In our more high-powered Experiment 3, the patterns were the same (Fig 1, third panel). In the less affluent treatment, the principal responded 29.1% of the time; in the more affluent treatment, the principal responded to 29.6% of emails. Again, this difference was substantively small and statistically insignificant (*p* = 0.88). In Experiment 4, where the statistical power was even higher and the treatment administered to a higher proportion of the sample with a greater treatment intensity, the response rates were also statistically and substantively equal. Those in the more affluent condition received responses from 9.4% of the sample; those in the less affluent sample received responses from 10.3% of the sample. This difference was substantively small and was not statistically significant (*p* = 0.39).

The same was true when we pooled the results from our experiments (Fig 1, far right panel). Our pooled estimates include a study fixed effect. (This does little to change the results.) Altogether, the emails in our less affluent treatment received responses about 16.6% of the time, those in our more affluent treatment received responses about 15.8% of the time, this substantive small difference was not statistically significant (*p* = 0.49), and the 95% confidence intervals were precise enough to allow us to rule out anything larger than a 1.32-percentage point bias in favor of one class or another. Our null effects are not for a lack of statistical power. To benchmark, our *upper bound effect* is only 26% of the *average* difference Butler and Broockman [12] observe across different racial treatments sent to state legislators (5.1 percentage points) and 14% of the social class gap that Butler [16] finds (9.6 percentage points).

Maybe, though, *how* public officials responded depended on the class of the person making the request. Answering this question is difficult due to the potential for post-treatment bias. To test this possibility, we examined the length of each reply [14] and whether officeholders provided the requested information or meeting. Results based on these measures (available on request) were once again null. In all of our experiments, we failed to find evidence of any social class bias in responses to simple casework requests.

Simply put, we find no evidence of class-based discrimination in our constituent request experiments regardless of sample, request, or treatment intensity.

## Discussion

In this paper, we presented the results from four audit studies seeking to ascertain the degree of social class bias in routine constituent service. The results of these experiments represent a significant departure from past research. In contrast to the literature on racial, religious, and age-based biases in constituent services, we find no evidence of a social class bias in how officeholders respond to the less affluent, regardless of whether the officials in question are elected state legislators and mayors or appointed public school principals. In contrast to research on unequal responsiveness in the policymaking process, we find no evidence that the affluent get preferential treatment in the basic casework process. And in contrast to the emerging idea that politicians give policy to the rich and services to the poor, we find no evidence that the less fortunate get preferential treatment. In our experiments, casework simply doesn’t seem to have much to do with social class.

Our work also shows compelling evidence that previously-identified racial biases[12] are unlikely to be driven by perceived differences in social class. Whereas public officials frequently discriminate in casework along racial/ethnic, religious, age, and gender lines, they do not seem to do so by class.

Of course, these experiments have limitations. Our work includes just a few kinds of public officials and cannot rule out discriminatory differences that are very small, or that might appear when citizens request even more burdensome services from officeholders, or that might appear when citizens convey social class in some other way beyond those that we have devised. Moreover, while our work suggests that politicians do not exhibit social class biases when they *answer* casework requests, there may still be biases in who *asks* for casework [23, 24]. And, of course, casework is no substitute for public policy, which is the subject of most research on unequal responsiveness. Our work makes an important contribution—to date, there have been few experiments on class biases in casework—but a great deal more could still be done.

## Supporting information

S1 Appendix Table A: Experiment 1 Email Text, Table B: Experiment 2 Email Text, Table C: Experiment 3 Email Text, Table D: Experiment 4 Email Text.
(DOCX)
