# Supplementary material for: Do public officials exhibit social class biases when they handle casework? Evidence from multiple correspondence experiments
Source: PLoS One. 2019 Mar 27;14(3):e0214244. doi: 10.1371/journal.pone.0214244 (PMC6436734; doi:10.1371/journal.pone.0214244)
Supplement: S1 Appendix — Table A: Experiment 1 Email Text, Table B: Experiment 2 Email Text, Table C: Experiment 3 Email Text, Table D: Experiment 4 Email Text. (DOCX) [file pone.0214244.s001.docx]

| Dear [Representative/Senator] [legislator’s name],  My name is Joey, and I am [**a dishwasher / an HR professional**]. I’m trying to figure out how to register to vote for the upcoming election. I heard that the voter registration deadline is soon.   Who should I call in order to register? Also, is there anything special I need to do when I register so that I can vote in future elections?   Thanks,  Joey |
| --- |

| Dear [Representative/Senator] [legislator’s name],  My name is Joey, and I am [**a dishwasher** **/ an HR professional**]. I’ve been following the recent debates about voter registration and House Bill 351, and I wanted to share my opinion with you.  Do you have any time in the next couple of weeks to meet or speak on the phone briefly?  Thanks, Joey |
| --- |

| Dear [Mr./Ms.] [Principal’s Last Name],  My name is Jessica. I'm emailing you on behalf of my son Joseph. My family is thinking about moving soon and would like to know more about your school. Specifically, I would like to know what music and art programs your school offers.  [**To share a bit about our family, we have struggled financially for the past few years. We've been on food stamps and Joseph has had to receive free/reduced lunches at school**.]  I'm emailing a few other schools in your area to see what they have to offer as well. I'd really appreciate hearing from you.  Thanks again,  Jessica |
| --- |

| To whom it may concern,  My name is Joey, and I am a [[**insert rich or poor profession**]] here in town. I'm emailing you because I’m planning to do some landscaping work, but I wanted to know who I should call before I dig.   Is there a town office that I should call? I’d really appreciate hearing from you.   Thanks, Joey |
| --- |
